# Enhancing ERP Responsiveness Through Big Data Technologies: An Empirical Investigation

**DOI:** 10.1007/s10796-023-10374-w

**Published:** 2023-02-18

**Authors:** Florie Bandara, Uchitha Jayawickrama, Maduka Subasinghage, Femi Olan, Hawazen Alamoudi, Majed Alharthi

**Affiliations:** 1 School of Business and Economics, Loughborough University, Loughborough, LE11 3TU UK; 2 Faculty of Business, Economics and Law, Auckland University of Technology, 120 Mayoral Drive, Auckland, New Zealand; 3 Essex Business School, University of Essex, Southend-On-Sea, SS1 1LW UK; 4 Marketing Department, College of Business, King Abdulaziz University, P.O. Box 344, Rabigh, 21911 Saudi Arabia; 5 Finance Department, College of Business, King Abdulaziz University, P.O. Box 344, Rabigh, 21911 Saudi Arabia

**Keywords:** ERP systems, ERP responsiveness, Big data technologies, Systematic literature review, Structural equation modelling

## Abstract

Organizations are integrating big data technologies with Enterprise Resource Planning (ERP) systems with an aim to enhance ERP responsiveness (i.e., the ability of the ERP systems to react towards the large volumes of data). Yet, organizations are struggling to manage the integration between the ERP systems and big data technologies, leading to lack of ERP responsiveness. For example, it is difficult to manage large volumes of data collected through big data technologies and to identify and transform the collected data by filtering, aggregating and inferencing through the ERP systems. Building on this motivation, this research examined the factors leading to ERP responsiveness with a focus on big data technologies. The conceptual model which was developed through a systematic literature review was tested using Structural equation modelling (SEM) performed on the survey data collected from 110 industry experts. Our results suggested 12 factors (e.g., big data management and data contextualization) and their relationships which impact on ERP responsiveness. An understanding of the factors which impact on ERP responsiveness contributes to the literature on ERP and big data management as well as offers significant practical implications for ERP and big data management practice.

## Introduction

By 2023, 65% of organizations will use enterprise resource planning (ERP) systems which are data centric and AI driven (Gartner, 2020). Organizations utilize ERP systems to manage and execute numerous business processes, which ultimately generate large volumes of data (i.e. big data) (Elragal, 2015). Big data is referred to as the increased volume of data that are difficult to store, process and analyze through traditional database technologies (Hashem et al., 2015; Saxena, 2016; Shi & Wang, 2018). Big data can be used as an enabler of novelty in the context of ERP systems, which helps organizations to streamline business processes in order to maximize the profitability (Chawda & Thakur, 2016; Marr, 2019), and gain competitive advantages (Jayawickrama et al., 2016; Jayawickrama & Yapa, 2013). Big data implementations support decision making and increases the ability to predict, which results in better financial performance and higher market values (Gupta et al., 2018; Huang et al., 2020). The use of big data enables effective resource allocation and enhances productivity, which ultimately bring competitive advantages for organizations (Chen et al., 2015).

The big data collected through ERP systems should be managed and transformed into meaningful knowledge, so that the organizations can make use of the big data to gain benefits. Organizations are making the use of big data technologies such as Apache Hadoop, R and NoSQL to support the big data management. Through the big data technologies, a wide-range of analytical functions can be executed on the big data collected through ERP systems, which can enhance the understanding of the business functions and increase the predictability (Fan & Perros, 2014; Infotech, 2019). For example, Zalando Payments GmbH (ZPS), a payment services provider for fashion retailer Zalando SE has integrated big data with ERP systems to produce reports (e.g. customer cash-in and factoring cash-outs) and provide employees real-time access to those reports (Schoenborn, 2021). This has increased the process efficiency, operational visibility and business growth of the organization (Schoenborn, 2021).

However, the organizations are struggling to manage the integration between ERP systems and big data technologies (Chokshi, 2020). For example, it is challenging for the organizations to manage large volumes of data collected through big data technologies and to identify and transform the collected data by filtering, aggregating and inferencing through the ERP systems. Furthermore, many organizations use only 12% of the collected data, leaving 88% of the data wasted (Chokshi, 2020; Joshi, 2019). Some of the reasons for those struggles include lack of managerial skills and technical skills required for big data technologies and organizations not having data-driven organizational cultures (Gupta et al., 2018). As a result, the organizations lack the ERP responsiveness, i.e., the ability of the ERP systems to react towards the large volumes of data that are been collected and processed while handling transactions and functionalities. Due to lack of ERP responsiveness, organizations are not realizing the benefits of big data (Chokshi, 2020). Thus, improving the ERP responsiveness can enhance the data utilization, while minimizing the data waste (Chokshi, 2020). Moreover, enhancing the ERP responsiveness results in understanding customer preferences, providing business insights, forecasting sales and improving supply chain management (Joshi, 2019).

We believe that the ERP responsiveness can be enhanced through; 1) big data management—managing the large amounts of data collected through ERP systems (Cui et al., 2020; Eine et al., 2017), and 2) data contextualization—identifying and transforming the data collected by filtering, aggregating, and inferencing through the ERP systems (Babu & Sastry, 2014; Gupta et al., 2018). It is important to understand the factors leading to big data management and data contextualization (Surbakti et al., 2020), so that the organizations can enhance big data management and data contextualization, which ultimately enhance the ERP responsiveness (Babu & Sastry, 2014; Eine et al., 2017). However, there is lack of research which explains the factors influencing big data management and data contextualization, and the relationship between big data management, data contextualization and ERP responsiveness. Thus, the two research questions that we aim to address are:

- *RQ1: what are the factors influencing big data management and data contextualization?*
- *RQ2: what is the relationship between big data management, data contextualization and ERP responsiveness?*

To examine our research questions, we conducted a two-phase analysis: phase 1 – systematic literature review (SLR) to identify the factors influencing big data management and data contextualization and to identify the relationship between big data management, data contextualization and ERP responsiveness, and phase 2 – quantitative survey to test the findings of phase 1.

This research is particularly noteworthy for three reasons. Firstly, it explains the relationship between big data management, data contextualization and ERP responsiveness. Although previous research has discussed the relationships between ERP systems and big data management (Haug et al., 2009; Jayawickrama et al., 2019), research which explains the management and the use of big data to enhance the ERP responsiveness are scarce. Secondly, previous research (Gupta et al., 2018; Huang & Handfield, 2015) has explained some factors influencing big data management and data contextualization. Yet, research which systematically identifies the factors influencing big data management and data contextualization are rare. Using a SLR, this paper identifies the factors influencing big data management and data contextualization. Thirdly, the model developed through this study will be helpful for managers in understanding the relationships between ERP systems and big data management. Furthermore, the model can be used as a guidance to enhance ERP responsiveness, which ultimately may minimize ERP and big data integration failures.

The rest of the paper proceeds in the following manner. Section 2 of the paper includes the research methodology. The subsequent section (i.e., Section 3) explains phase 1: process and results of SLR. The literature review of this research is presented through the SLR. This section highlights the research gaps and develops a conceptual model. This is followed by Section 4 which explains phase 2: empirical data collection, analysis and results. The conceptual model developed through the phase 1—SLR was validated through phase 2—empirical data collection and analysis. Next, the paper includes Section 5: the discussion section. This is followed by Section 6 which includes theoretical and practical implications. The paper concludes with Section 7, which includes limitations and future research.

## Research Methodology

This research was conducted in two phases: phase 1—SLR, where secondary data was gathered from existing scientific sources, and phase 2—the quantitative phase in which empirical data was gathered and analyzed using statistical formulae. SLR allows researchers to identify and understand findings of various other researchers who have previously explored a branch or the entirety of the chosen research area (Kupiainen et al., 2015; Pacheco et al., 2018). Thus, the ultimate goal of phase 1- SLR was to understand the relationship between big data and ERP systems through the analysis of previous research. SLR was conducted in four steps: identification—plan of the research was reviewed, and the research questions were identified, collection – articles screened on the basis of the title and abstract, analysis – full-text articles assessed for eligibility and process – studies included in the qualitative synthesis. Phase 1 resulted in a conceptual model and initial hypotheses, which indicated the possible relationships between the identified variables of the conceptual model.

During phase 2: the conceptual model which was developed through the SLR was tested using Structural equation modelling (SEM) performed on the survey data collected from 110 industry experts. Previous research (Askool & Nakata, 2011; Bukhari et al., 2013; Whyte & Lamprecht, 2013) has explained that quantitative method is appropriate for validating conceptual models which consist of the constructs and relationships derived through the existing literature. This is because one of the aims of using quantitative method is to test causal relationships between variables (Pinsonneault & Kraemer, 1993). Some examples of the application of quantitative method to validate conceptual models in related fields include Wu and Chen (2005), Hassandoust et al. (2022) and Chau (1996). During phase 2, quantitative data was collected from industry experts who were knowledgeable on ERP systems and big data technologies using an online self-administered questionnaire. The survey was developed using previously validated items and refined through a pilot study before sharing with the actual participants. The survey provided the authors the opportunity to present standardized questions to all the participants involved, collect a substantial volume of data in a short time frame and to facilitate the data analysis in a systematic and a quantifiable manner. Surveys provide the ability to identify the common relationships across multiple organizations, thus provide generalizable results (Gable, 1994). Moreover, surveys are appropriate when the researchers have clearly defined dependent and independent variables and expected relationships and attempt to test those variables and relationships (Pinsonneault & Kraemer, 1993). In this research we have developed a conceptual model with a clear indication of independent and dependent variables, thus the survey method is appropriate to test the conceptual model of our study (Askool & Nakata, 2011; Bukhari et al., 2013; Whyte & Lamprecht, 2013). We were able to collect 110 complete responses for the survey, which were then analyzed through statistical techniques. The following section explains the process and results of phase 1 – SLR, whereas Section 4 explains phase 2—quantitative study in-detail.

## Phase 1 – Process and Results of SLR

Sections 3.1 and 3.2 explain the process of SLR and the results of SLR respectively.

### Process of SLR

**Fig. 1:**
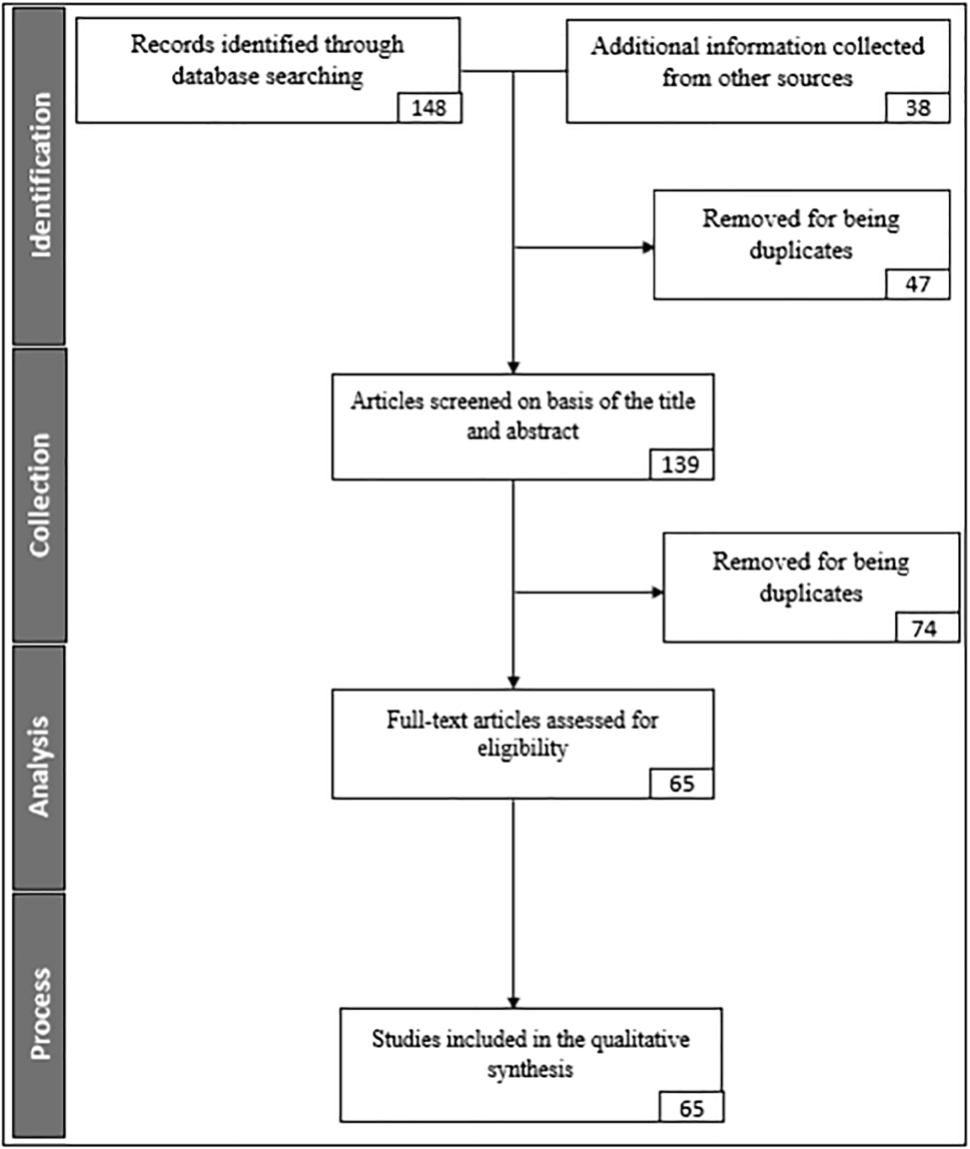
SLR Framework

Following Saunders et al. (2012) and Kitchenham and Charters (2007) methodological guidelines, the systematic literature review was conducted in four steps: identification, collection, analysis and process as shown in Fig. 1.

To adhere to the best practices of conducting a systematic literature review, the authors established the following criteria when selecting literature to be reviewed:

1) journal articles, conference proceedings and recommended book chapters related to the research topic published on scientific databases must be considered – Following this criterion, the scientific literature for the purpose of conducting the systematic literature review was obtained through scientific databases and search engines such as Google scholar, Scopus and ACM digital library. When conducting a SLR, it is a must to explicitly define the search boundaries to ensure the quality of appraisal aligned with the research scope (Saunders et al., 2012). To identify suitable literature, we have used the following search query; “ERP systems” AND “big data technologies” AND “ERP innovations” AND “ERP data management” AND “unambiguous data management”, 2) all literature to be analyzed in the SLR were published within the timeframe of active period of interactions between big data and ERP systems. Thus, all scientific literature published within the timeframe of 2012 - 2020 were considered, with exceptions for regularly updated webpages of leading computing research organizations and computing technology organizations, 3) the literature exploring the research topic from any geographical region were considered, 4) qualitative and quantitative studies were reviewed including conceptual papers, and 5) only literature published in the English language were chosen.

In the identification step, plan of the research was reviewed, and the research questions were identified. As we wanted to identify the papers which are highly relevant to our study, we have used the following search query; “ERP systems” AND “big data technologies” AND “ERP innovations” AND “ERP data management” AND “unambiguous data management”. As a result, we were able to identify 148 papers which are highly relevant to our study. The total of 148 literature was categorized in to three themes: theme 1—correlation between ERP systems and big data – i.e. the connection between ERP systems and big data (50 papers), theme 2 – impact of big data technologies on ERP systems – i.e. the influence of big data on ERP systems (56 papers), and theme 3 (42 papers)—how the correlation between ERP systems and big data technologies affects different industries – i.e. industry specific characteristics of the connection between ERP systems and big data. Thereafter, a total of 38 papers were identified from other sources performing forward and backward search, making the total number of papers to 186. Those 38 papers were also assigned to the three categories, 13, 10 and 15 respectively. Among the 186 papers, 47 papers were removed for being duplicates (for example, conference papers were removed where journal articles were published based on those conference papers). There were 20 duplicates in theme 1, 17 in theme 2 and 10 in theme 3. Out of the remaining 139 papers, 74 papers were removed from the analysis due to reasons such as being irrelevant to the phenomenon of study, lack of clarity, complicated nature of the findings. This resulted in final count of the papers which can be used for analysis as 65, out of those 65, 13 belongs to theme 1, 25 belongs to theme 2 and 27 belongs to theme 3. The SLR process was finalized by creating a conceptual model and initial hypothesis, which were then tested through phase 2 of the study (see Section 4). Table 1 depicts the results after conducting the SLR of this study.

**Table 1:** Results after conducting the SLR

Steps	Total	Theme 1: Correlation among ERP and big data	Theme 2: Impact of big data technologies on ERP systems	Theme 3: The means in which the correlation of ERP systems and big data technologies affect in specific industry sectors
Identification: initial	148	50	56	42
Total	148			
Identification: addition of other sources	*38*	13	10	15
Total	186	63	66	57
Identification: removed for being duplicates	*47*	20	17	10
Total	139	43	49	47
Collection: removed by reading the content	*74*	30	24	20
Studies included in the qualitative synthesis	65	13	25	27

**Fig. 2:**
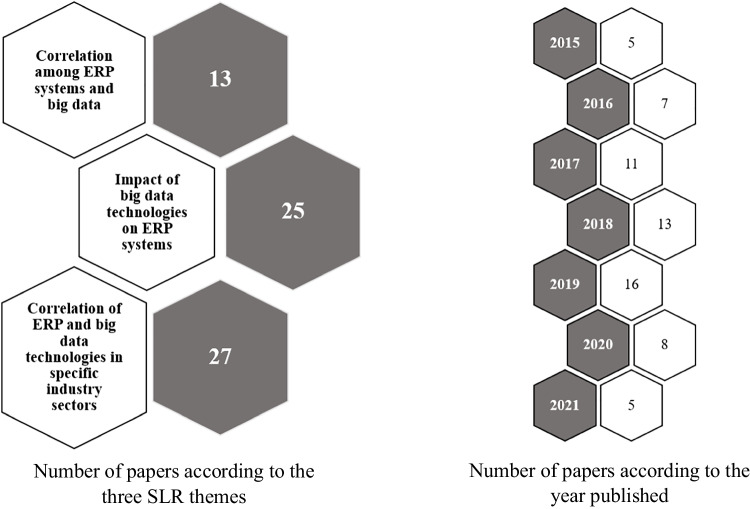
Illustration of chosen papers according to the year published and the three themes

The following two graphs (Fig. 2) illustrates the studies included in the qualitative synthesis according to the three themes and the year published.

### Results of SLR

The SLR was helpful not only to identify the factors influencing big data management, data contextualization and ERP responsiveness but also to improve our understanding on the ERP systems, application of big data technologies, integration of ERP systems with big data technologies, issues in ERP responsiveness, issues in big data management and issues in data contextualization in general. The SLR allowed the authors to gain insights into solutions developed by the other researchers, which were aimed at improving big data management, data contextualization and ERP responsiveness.

#### ERP systems

According to Accenture group (Ellingsen, 2018), more than 67% of the renowned companies have adopted ERP systems. 35% of companies out of the 67% are currently using on-premises ERP systems, while 12% are hybrid. The survey carried out by the ERP buyer’s profile for growing companies predicts that by the next 3–5 years; at least 53% of the companies worldwide will move to cloud ERPs completely (Mintchell, 2018). ERP systems seamlessly integrate different business processes across the departments and functional areas into a centralized system (Davenport, 1998; Hashem et al., 2015; Jayawickrama et al., 2017; Kim et al., 2018; Mahmood & Lloyd, 2017; Saxena, 2016; Shi & Wang, 2018; Wagner et al., 2004). Those systems connect people, processes, data and things in an intelligent and strategic manner that allows organizations to create value from data streams generated through ERP systems (Grover et al., 2018; Lehrer et al., 2018). ERP systems integrate various value chain activities, manage inventory, enhance enterprise visibility, provide operational excellence, improve customer relationship management and increase real-time information access, while reducing operational errors (Alkraiji et al., 2020; Ellingsen, 2018).

Previous research mainly focused on the implementation of ERP systems (Akkermans & van Helden, 2002; Al-Mudimigh et al., 2001; Chen et al., 2016; Jayawickrama et al., 2019; Monk & Wagner, 2012; TechTerms, 2020; Tsai et al., 2012), the ERP vendor selection (Wickman et al., 2018) and advantages and disadvantages of implementing ERP systems (Stefanou, 2001; Trimi et al., 2005). However, research which specifically focuses on the relationship between big data and ERP systems are scarce (Tsai et al., 2012).

There is a specific set of features to be considered in implementing ERP systems in each industry. Thus, ERP systems should be customized as per the requirements of each industry, or the business processes of the industry should be altered as per the functionalities of the ERP systems. Companies in various industries, which are sharing the common goal of developing innovative technologies, are starting to realize the benefits of big data technologies and the importance of integrating big data technologies with ERP systems (Elragal, 2015; European Commission, 2019).

#### Application of Big Data Technologies

Organizations are facing complex and competitive environment than ever before (Tenhiälä et al., 2018; Yeow et al., 2018; Zong et al., 2017). Business success is no longer a fact of only centralizing the business functionalities, but rather the managing of large amounts of frequently collected data (Agarwal & Dhar, 2014; Jayawickrama et al., 2016; Li et al., 2019; Lorenc, 2015). Big data technologies are evaluated by the concept of the 8 V’s which stands for volume, value, veracity, visualization, variety, velocity, viscosity and virality (Badea et al., 2018; Deloitte, 2015; Marr, 2019). Chawda and Thakur (2016) states that more than 90% of data in the world has been created within the last two years. Mattews (2018) describes that most of the data collected in the world are by the 220 millions of self-driven cars, which automates the functions by use of information systems and machine learning techniques. Moreover, the big data market expects to grow in 20% every year after 2019 (Badea et al., 2018).

Big data generated through various mediums (e.g., social media, web searches, smart watches and customer tracking using business intelligence) can be used for predictions, so that business processes can be optimized (Bekker, 2018; Kim et al., 2018; Marr, 2019; Rastogi, 2018; Wickman et al., 2018). For example, the big data generated through social media interactions can be used to predict future buying intensions of the individuals. Thus, the organizations can use this data to predict future sales and optimize their business processes. The internet and the new technologies are challenging the traditional data structures that firms have adopted to handle various business functionalities (Gill, 2017). Big data can help organizations to be more transparent, satisfy customer requirements in a customized manner and keep up with volatile market conditions (Davenport, 1998; Deloitte, 2015; Gupta et al., 2018; Müller et al., 2018). With the development of business trends; it is quite evident that most of the data collected through various means are fallen in to the category of big data, resulting in enhancing business processes, business performance optimization, improving machine and device performance and financial trading (Bekker, 2018; Grover et al., 2018; Marr, 2019; Rastogi, 2018; Saxena, 2016; Vaghela, 2018).

Big data is an emerging area in various industry sectors (Rastogi, 2018). For example, big data performs a vital role in financial trading sector by execution of high frequency trading (Infotech, 2019; Marr, 2019; Mattews, 2018). Various industries use data algorithms in business decisions making, which affects current and future business performance (Shi & Wang, 2018).

#### Integration of ERP Systems with Big Data Technologies

It is challenging to collect and process large amount of structured and unstructured data collected through ERP systems (Saxena, 2016). Saxena (2016) considers ERP systems as a data bank, which is not capable of handling big data. The big data technologies which were mainly used to understand the data collected through social networking sites, are now being used to understand the data collected through ERP systems (Akhtar et al., 2017; Jayawickrama & Yapa, 2013; Lorenc, 2015; Saxena, 2016; Shi & Wang, 2018; Wickman et al., 2018). Organizations are collecting and analysing big data with the intension to enhance the efficiency of ERP systems (Jayawickrama & Yapa, 2013; Jayawickrama et al., 2016). While big data technologies do not alter the functionality or the methods used by ERP systems (Haug et al., 2009; Jayawickrama et al., 2019), it enhances sales forecasts, scheduling and supply chain management (Saxena, 2016). Most organizations rely on ERP systems as they do not act just as data repositories, but as smart systems which collect, analyze and predict the future of the business with use of the big data technologies. Integration of ERP systems with big data has become a core factor in most of the industries. For a proper integration of ERP systems with big data technologies, there should be a solid management of organizational structures as well as processes (Deloitte, 2015).

#### Issues in ERP Responsiveness

Improving the ERP responsiveness is challenging due to the increased complexity of business processes and extended supply chains (Carr, 2016; Fox, 2015; Gill, 2017; Plex, 2014). The present situation of the ERP systems is quite different to ERP systems used before (Mahmood & Lloyd, 2017), as the current ERP systems demand new levels of collaboration throughout the supply chains, inside and outside the enterprises (Gill, 2017). Thus, it is challenging for the organizations to react towards the vast amount of data collected through ERP systems. Improper management of data increases the complexity of data manipulation, which ultimately minimizes responsiveness and mobility of ERP systems (Chawda & Thakur, 2016; Jayawickrama & Yapa, 2013; Tenhiälä et al., 2018; Zong et al., 2017). Lack of ERP responsiveness and mobility (Cole, 2018; Jayawickrama et al., 2016; Shi & Wang, 2018) affect on the entire business process starting from decision making to the profit gaining (Saxena, 2016).

#### Issues in Big Data Management

Data is the core of each and every decision-making process (Elragal, 2015; Lin et al., 2016; Wickman et al., 2018), thus it is important to collect and analyze data, so that the organizations can make informed decisions. Large amounts of data collected through different processes have been used to support various business activities such as business process optimization, business performance optimization, machine and device performance optimization and financial trading (Bekker, 2018; Marr, 2019; Vaghela, 2018). The use of inefficient data management systems can lead to inaccurate business decisions (Saxena, 2016) such as inaccurate estimations of customer preferences (Lorenc, 2015). Improper data management may cause exceeded data storage (Marr, 2019) by collecting information on unnecessary transactions (Bekker, 2018; Vaghela, 2018). The exceeded data storage and lack of appropriate data management techniques can affect data management capacities of the organizations, ultimately leading to high expenses (Shi & Wang, 2018; Voigt et al., 2016).

#### Issues in Data Contextualization

ERP systems integrate data generated through various departments into a centralized database, allowing the system to generate business reports (Nah & Delgado, 2006). ERP systems no longer act as a data repository, but as an analytical repository which monitor manufacturing processes, manage communication channels, analyze electronic documents and optimize inventory management and finance management (Mahmood & Lloyd, 2017). Through the transaction processing and workflow management functions, ERP systems allow companies to gain an overall understanding of the business process and enhance the data analysis capabilities (Voordijk et al., 2003). The business gains are only possible if the organizations can analyze and make sense of real-time up-to-date large volumes of data generated through ERP systems (Mabert et al., 2003). However, analysis of large volumes of data generated through ERP systems is challenging. Therefore, companies are using big data technologies to support the data analysis.

#### Formulation of the Conceptual Model and the Hypotheses

**Fig. 3:**
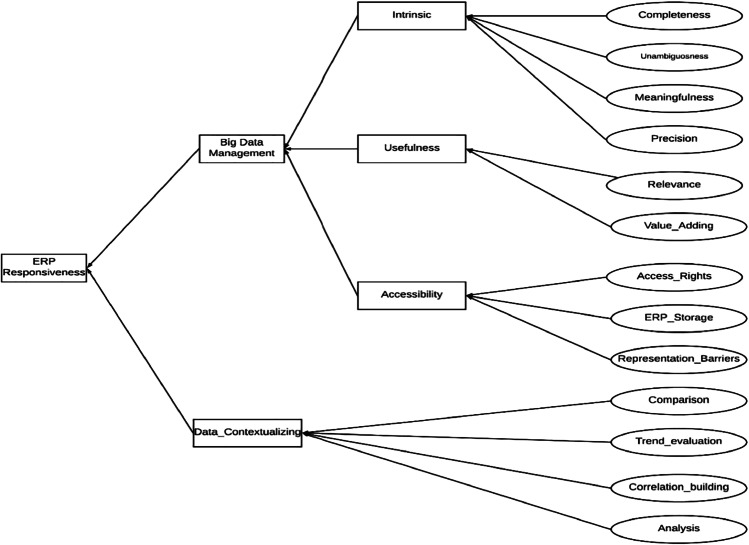
Conceptual Model

The goal of the research was to examine the factors influencing big data management and data contextualization and the relationship between big data management, data contextualization and ERP responsiveness. Thus, the research model encompasses and relies on three areas; 1) big data management as in articles explained by Jayawickrama et al. (2016), Mahmood and Lloyd (2017), Simon (2017) and Bekker (2018), 2) data contextualization as explained by Fox (2015), Elragal (2015) and Li et al. (2019), and 3) ERP responsiveness based on Chokshi (2020), Babu and Sastry (2014) and Eine et al. (2017); Fox (2015). We developed the conceptual model based on the selected literature on ERP systems and big data technologies (see Fig. 3).

Technological integration of ERP systems with big data technologies result in improvised and managed data, paving main means to solve issues related to ERP responsiveness. Issues related to big data management can be minimized by the intrinsic ability, usefulness and accessibility (Bekker, 2018; Infotech, 2019; Jayawickrama et al., 2016; Simon, 2017; Solutions, 2018).

##### Intrinsic Factors

Intrinsic factors are the most simplistic and the most essential elements in big data management (Hashem et al., 2015). Intrinsic factors are mainly influenced by four variables, i.e. completeness (Hashem et al., 2015; Shi & Wang, 2018; Wickman et al., 2018), unambiguousness (Deloitte, 2015; Hashem et al., 2015), meaningfulness (Orosz & Orosz, 2014; Yaqoob et al., 2016), and precision (Liu et al., 2019; Voigt et al., 2016). Completeness means the data collected through ERP systems are complete, where there is lack of missing data. The specific information requirements of the organizations can be achieved when the data collected through ERP systems are complete (Wickman et al., 2018). Comprehensiveness of available data in the ERP system improves data integrity (Wickman et al., 2018).

ERP systems perform various business functions in a centralized environment. The performance of organizations can be enhanced when the data generated by ERP systems are unambiguous (Hashem et al., 2015). Unambiguousness is defined as the data collected through ERP systems are clear and concise, thus the data are not open to more than one interpretation. Unambiguous data can maximize the performance of the organizations. Meaningfulness of the data play a pivotal role in big data management (Hashem et al., 2015; Wickman et al., 2018). Meaningfulness is defined as the data collected through ERP systems have a great value or significance. It is vital that the organizations enhance the meaning of each and every module in the ERP system by connecting the data to the relevant sections in ERP systems (Hashem et al., 2015). Precision of the data is crucial in decision making. Precision is defined as the data collected through ERP systems are exact and accurate. Similarly, precision of the big data may decrease confusions arisen when mapping data generated through ERP systems (Davenport, 1998). Thus, it is proposed;

- **H1a—**Intrinsic factor mediates positive effect of the completeness on big data management.
- **H1b—**Intrinsic factor mediates positive effect of the unambiguousness on big data management.
- **H1c—**Intrinsic factor mediates positive effect of the meaningfulness on big data management.
- **H1d—**Intrinsic factor mediates positive effect of the precision on big data management.

##### Usefulness Factors

Usefulness is the state of being useful or quality of the data collected by ERP systems (Hashem et al., 2015). Usefulness is influenced by two variables; 1) relevance – the level of appropriateness of the data collected and generated through the ERP systems (Badea et al., 2018; Gupta et al., 2018), and 2) value-adding—how much value is added to the data collected by ERP systems while increasing the validity of the data in order to improvise the usability (Spathis & Constantinides, 2003; Yaqoob et al., 2016). Usefulness of the data can be enhanced when the data collected through ERP systems are relevant and value adding. Useful data collected through ERP systems enhances the necessity of big data management. Thus,

- **H2a—**Usefulness mediates positive effect of relevance on big data management.
- **H2b—**Usefulness mediates positive effect of value adding on big data management.

##### Data Accessibility

Data accessibility means the quality data being able to be reached or entered. Poor data accessibility is a common issue faced by many organizations (Orosz & Orosz, 2014; Yaqoob et al., 2016). When the managers are provided with access to important information of a business such as costs and profits, they can obtain a complete understanding of the business, which enhances the ability to identify new opportunities and overcome the existing challenges (Infotech, 2019). The key variable accessibility is influenced by three variables as access rights (Calisir & Calisir, 2004; Schlichter & Rose, 2013; Zhezhnych & Tarasov, 2018), ERP storage (Ellingsen, 2018; Haug et al., 2009; Li et al., 2019) and representation barriers (Barth & Koch, 2019; Calisir & Calisir, 2004; Haug et al., 2009).

Access rights means providing the data access with user-based permission, thereby minimizing the possibility of security breaches and safeguarding sensitive data (Calisir & Calisir, 2004; Zhezhnych & Tarasov, 2018). By implementing access rights, the users can access and perform only the operations that they are allowed to, yet if required, the users can be given view only access as well. Monitoring access reviews, strong password hygiene, make use of identity tracking software are some of the measures to be taken when implementing access rights (Schlichter & Rose, 2013; Zhezhnych & Tarasov, 2018). ERP storage focusses on the storage space needed for an ERP system to securely store data and to which extent the users can guarantee on the security level of the storage (Haug et al., 2009; Li et al., 2019). ERP representation barriers explains about the display methodology used in presenting the data collected and analyzed by the ERP systems with use of the big data technologies (Ellingsen, 2018; Huang & Handfield, 2015). It is important to be aware how easy it is to display the necessary details clearly and concisely. Thus, it is proposed;

- **H3a—**Accessibility mediates positive effect of access rights on big data management.
- **H3b—**Accessibility mediates positive effect of ERP storage on big data management.
- **H3c—**Accessibility mediates positive effect of representation barriers on big data management.

##### Data Contextualization

As per Fox (2015), ERP responsiveness can be enhanced by data contextualization (Elragal, 2015; Li et al., 2019). Data contextualization is influenced by four factors: comparison (Chawda & Thakur, 2016; Haug et al., 2009; Jayawickrama & Yapa, 2013), trend valuation (Elragal, 2015; Li et al., 2019), correlation building (Akhtar et al., 2017; Jayawickrama et al., 2017; Lin et al., 2016), and analysis (Elragal, 2015; Jayawickrama & Yapa, 2013). Comparison is defined as comparing the relevant data in different ERP modules and reacting accordingly (Yaqoob et al., 2016). Trend evaluation is the ability to identify the trends by considering the data and fluctuations into consideration (Schlichter & Rose, 2013; Voigt et al., 2016). Correlation building is the ability to build up the causality among data generated through day-to-day operations of ERP modules (Zhezhnych & Tarasov, 2018; Zong et al., 2017). Analysis is defined as the ability of analysing the data collected using developed technologies to make timely and precise decisions.

- **H4a—**Data contextualization mediates positive effect of comparison on ERP responsiveness.
- **H4b—**Data contextualization mediates positive effect of trend evaluation on ERP responsiveness.
- **H4c—**Data contextualization mediates positive effect of correlation building on ERP responsiveness.
- **H4d—**Data contextualization mediates positive effect of analysis on ERP responsiveness.

##### Big Data Management and ERP Responsiveness

It is important to integrate the data generated through ERP systems with the data generated through legacy systems, so that the organizations can gain maximum benefits through ERP systems (Nah & Delgado, 2006). The transaction processing function of ERP systems allows integrated management of the data generated through various systems, so that the managers can gain an overall understanding about the business processes through the system (Voordijk et al., 2003). Inefficient data management lead to inaccurate business decisions and incorrect identification of customer preferences (Saxena, 2016). Moreover, the inefficient data management can lead to the collection of unnecessary data, which ultimately increases the expenses (Bekker, 2018; Vaghela, 2018). By properly managing large volumes of data generated through ERP systems, the managers can make informed business decisions as well as delegate authority to employees (Mabert et al., 2003). Thus, it is proposed;

- **H5—**Big data management is positively related with ERP responsiveness.

## Phase 2—Empirical Study

Section 4.1 explains empirical data collection whereas Section 4.2 discusses empirical data analysis and results.

### Empirical Data Collection

To empirically test the conceptual model presented in Fig. 3, we conducted a quantitative study in the form of an online self-administered questionnaire with industry experts in ERP systems and big data technologies (see Table 2 and appendix A for the operationalization information and questionnaire respectively). A five-point Likert scale (strongly disagree, disagree, neither agree nor disagree, agree and strongly agree) was used to measure all of these items.

**Table 2:** Table of operationalization

Hypothesis	Independent Variable	Dependent Variable	Questions	References
H1a	Completeness	Big data management	7, 8, 9	(Hashem et al., 2015; Shi & Wang, 2018; Wickman et al., 2018)
H1b	Unambiguousness	Big data management	10, 11	(Deloitte, 2015; Hashem et al., 2015)
H1c	Meaningfulness	Big data management	9,11,12	(Orosz & Orosz, 2014; Yaqoob et al., 2016)
H1d	Precision	Big data management	13, 14	(Liu et al., 2019; Voigt et al., 2016)
H2a	Relevance	Big data management	15, 16	(Badea et al., 2018; Gupta et al., 2018)
H2b	Value adding	Big data management	16, 17	(Spathis & Constantinides, 2003; Yaqoob et al., 2016)
H3a	Access rights	Big data management	18, 19, 20	(Calisir & Calisir, 2004; Spathis & Constantinides, 2003; Zhezhnych & Tarasov, 2018)
H3b	ERP storage	Big data management	21, 22, 23	(Ellingsen, 2018; Haug et al., 2009; Li et al., 2019)
H3c	Representation barriers	Big data management	23, 24	(Barth & Koch, 2019; Calisir & Calisir, 2004; Haug et al., 2009)
H4a-d	Data contextualization	ERP responsiveness	26, 31	(Tenhiälä et al., 2018; Zong et al., 2017)
H4a	Comparison	ERP responsiveness	26, 27	(Chawda & Thakur, 2016; Haug et al., 2009; Jayawickrama & Yapa, 2013)
H4b	Trend evaluation	ERP responsiveness	28, 29	(Elragal, 2015; Li et al., 2019)
H4c	Correlation building	ERP responsiveness	30, 32	(Akhtar et al., 2017; Jayawickrama et al., 2017; Lin et al., 2016)
H4d	Analysis	ERP responsiveness	31, 32	(Elragal, 2015; Jayawickrama & Yapa, 2013)
H5	Big data management	ERP responsiveness	25, 32	(Zhezhnych & Tarasov, 2018; Zong et al., 2017)

Two eligibility questions were included to assess the participant’s awareness on ERP systems and big data technologies, so that they can decide if they are eligible to participate in the survey (i.e., 1.I have worked and/or have a sound knowledge on Enterprise Resource Planning (ERP) systems and 2. I have worked and / or have a sound knowledge on big data technologies). The survey questions were refined through logical validity technique and test–retest reliability technique through a pilot study, which was conducted with a set of industry experts. The survey link of the actual study was shared in three steps: step1 – published among the social media networks such as LinkedIn, step 2—published on the user groups related to ERP systems and big data technologies, and step 3—the link of the questionnaire was sent via email, WhatsApp messages and was posted on Facebook user groups of the pool of the participants identified via the participants of the pilot study (i.e. through snowball sampling method (Minichiello, 1995)).

A total of 561 individuals were connected via LinkedIn, where the questionnaire was mainly published. In other platforms where the questionnaire was published, there were large number of active and inactive users, thereby making it difficult to calculate the accurate response rates for the data obtained through those sites. Therefore, we calculated the response rate only from the 561 individuals who were connected through LinkedIn messages. Out of the 561 individuals, 110 individuals responded to the questionnaire. Thus, the response rate for the questionnaire was approximately 19.8%, which is considered a good response rate for a quantitative analysis (Saunders et al., 2012). Out of the sample of 561, 63 responders were not familiar with big data technologies, despite of the fact that them being ERP experts. Moreover, 32 responders could not answer the questionnaire as the participants were lacking the experience on ERP systems, whilst working with big data technologies.

### Empirical Data Analysis and Results

This section explains the results of descriptive analysis and structural equation modelling (SEM).

#### Descriptive Analysis

A majority of the participants were employed in organizations with more than 250 employees (60.6%), which had an annual turnover of more than 61 million USD (54.3%) (See Table 3, Fig. 2). As per the European Commission’s definition of company categorization (European Commission, 2019), companies with more than 250 staff or companies with an annual turnover of more than or equal 50 million Euros can be considered as large size organizations (50 million Euro ≅ 61 million USD). Therefore, it can be concluded that most of the participants were employed at large organizations. However, our sample included participants from the small and medium size organizations as well, which indicates that not only the large organizations, but also the small and medium size organizations have adopted ERP systems along with big data technologies. Majority of the participants (i.e., 40.4%) had more than 11 years of experience in ERP and big data domains.

**Table 3:** Demographic information of the participants

Number of employees	Percentage
< 50	9.6%
< 250	29.8%
> 250	60.6%
Annual turnover	
< 12 million USD	23.4%
< 61 million USD	22.3%
> 61 million USD	54.3%
Number of years of experience in ERP and big data domains	
0–2	13.8%
2–5	20.2%
5–7	12.8%
7–10	12.8%
> 11	40.4%

SAP was the most used trademark of ERP systems with a 28.7% of the total number of participants, whereas the second popular trademark of ERP systems was Oracle with 25.5%, see Table 4. As per Magal (2012), if the entire company’s business processes are automated, it is more likely that particular company to use SAP, Oracle or Microsoft Dynamics, while Law and Ngai (2007) clarifies that some companies only use certain modules of ERP systems by partnering with Sage, Infor, EPICOR.

**Table 4:** Information on ERP trademarks and big data technologies

ERP Trademarks	Percentage
SAP	28.7%
Oracle	25.5%
Microsoft Dynamics	12.8%
IFS Applications	8.5%
Sage	5.3%
Infor	2.1%
Epicor	4.3%
Other	12.8%
Big data technologies	
NoSQL	26.6%
Predictive Analytics	23.4%
Blockchain	20.2%
Apache Hadoop	19.1%
R	17.0%
MongoDB	12.8%
Apache Spark	5.3%
Cassandra	4.3%

Considering the big data technologies that are most capable in dealing with ERP systems, NoSQL take the lead, which is followed by predictive analytics, blockchain and Apache Hadoop (see Table 4). Consistency, availability and partition (CAP) theorem explains that consistency, availability and partition tolerance nature of NoSQL are the main causes for it being the most used big data technology in dealing with ERP systems (Ekren & Erkollar, 2020). Moreover, Smith (2012) mentions that as the ERP systems heavily rely on SQL databases, ERP systems require more consistency and transitioning functionalities. Thus, the ability of NoSQL as explained by the CAP theorem would help in easing the data analytics of ERP systems (Radulović et al., 2016; Saxena, 2016; Smith, 2012).

**Fig. 4:**
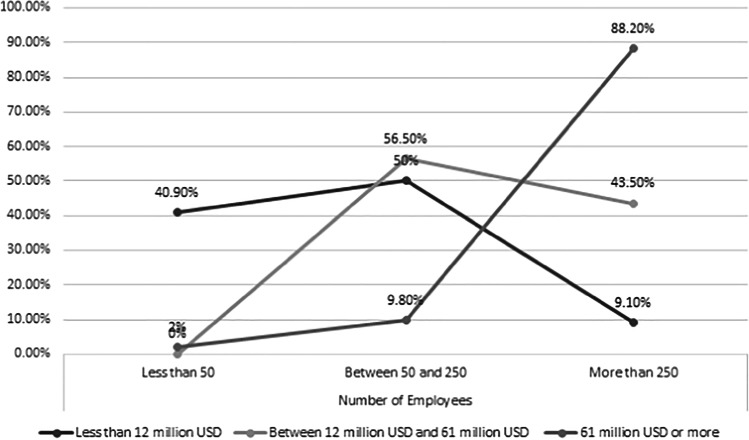
Information about the percentage of employees according to the annual turnover

Figure 4 depicts the percentage of employees divided according to the annual turnover of the companies in which they are employed. 40.9% of the companies which have an annual turnover of less than 12 million US dollars have less than 50 employees, whereas 2% of the companies that have an annual turnover of 61 million US dollars and more have less than 50 employees. Only 9.10% of the companies that have an annual turnover of less than 12 million US dollars have more than 250 employees, whereas 88.2% of the companies that have an annual turnover of 61 million US dollars and more have more than 250 employees. This indicates that companies which have an annual turnover of 61 million US dollars or more significantly use the interaction between ERP systems and big data technologies, whereas the companies with less than 61 million US dollars have a gradual growth of use of the interactions between ERP systems and big data technologies to gain competitive advantage through business optimization.

**Fig. 5:**
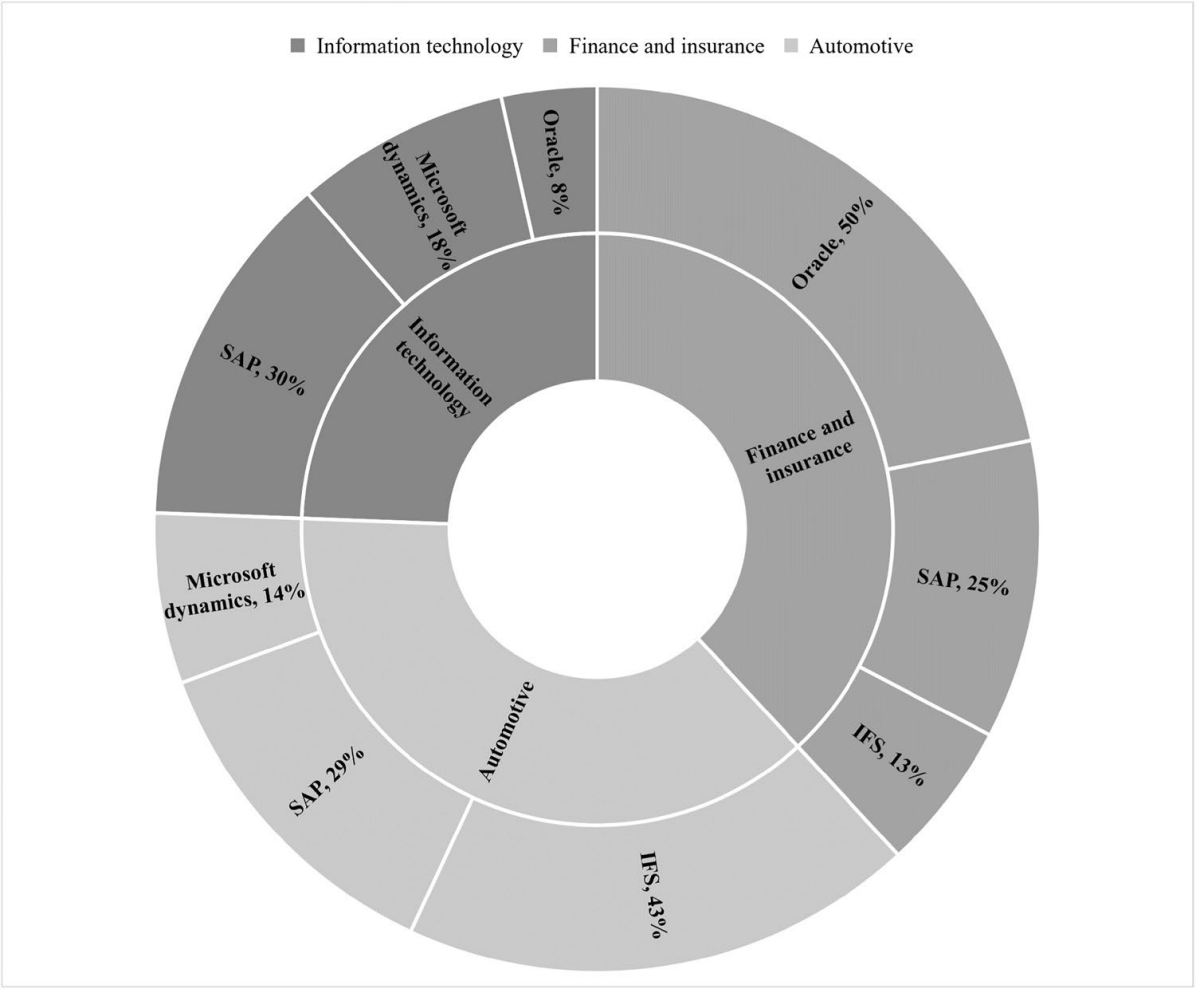
Top 3 ERP systems used by the top 3 industry sectors using the correlation between ERP systems and big data technologies

Figure 5 demonstrates the top 3 industry sectors and their most used ERP systems. It is conclusive that all the 3 industries (information technology, finance and insurance, and automotive industry) use SAP as the ERP system. The most used ERP system in the information technology industry sector is SAP (30%), while the most used ERP system in finance and insurance industry sector is Oracle (50%) and the automotive industry uses IFS (43%). It is evident that IFS is used in both automotive and finance and insurance sectors while Oracle used in both information technology and finance and insurance industry sectors. Moreover, Microsoft dynamics has also been commonly used in information technology as well as automotive sectors.

**Fig. 6:**
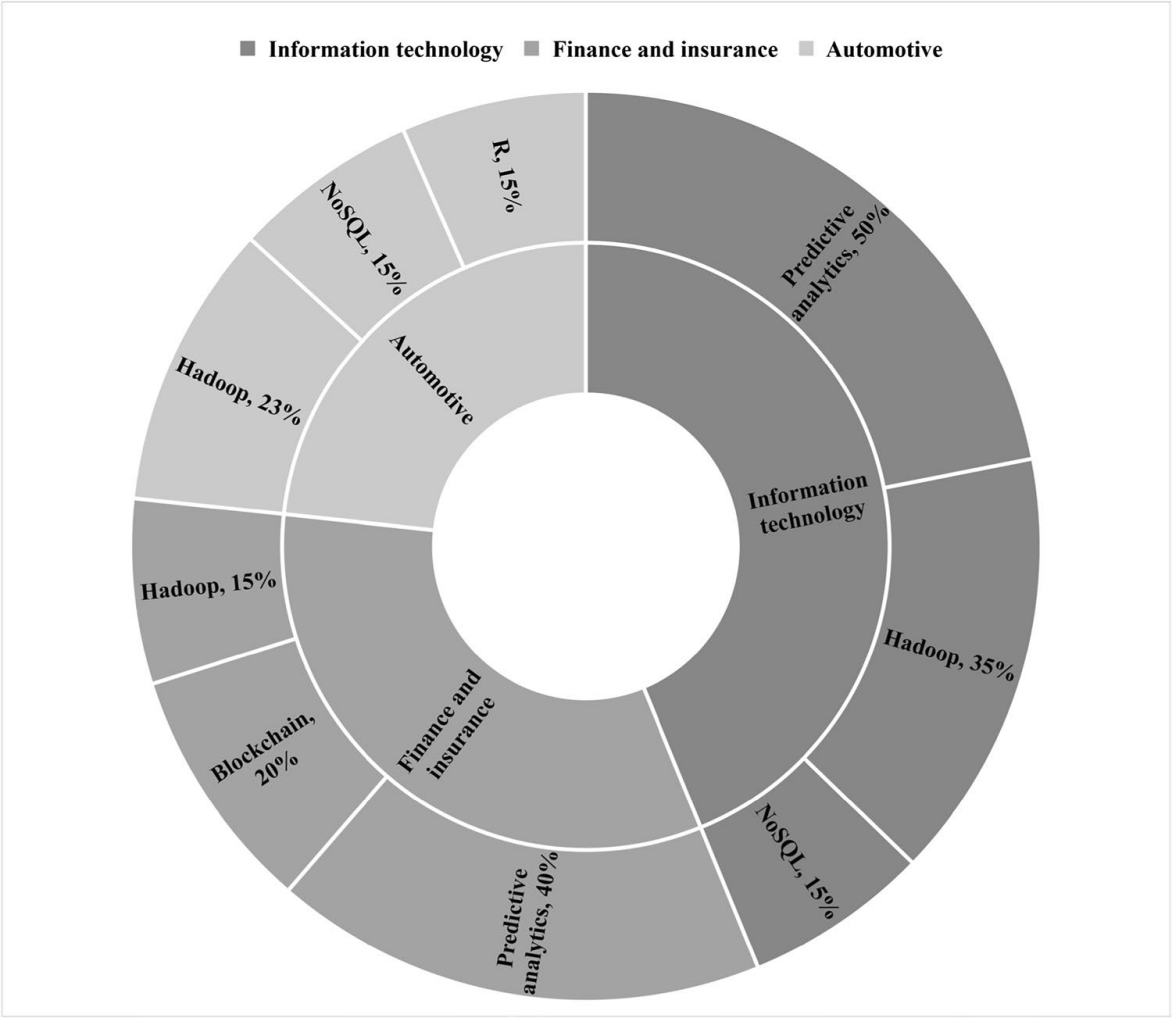
Top 3 big data technologies used by the top 3 industry sectors using the correlation between ERP systems and big data technologies

Figure 6 depicts the top 3 industry sectors and their 3 most used big data technologies. Hadoop is a big data technology used by all three industry sectors while predictive analytics is used in information technology and finance and insurance sectors. NoSQL is a commonly used big data technology in information technology industry sector as well as automotive industry. Apart from Hadoop and NoSQL, 15% of automotive industry uses R, while 20% of the finance and insurance uses blockchain.

Among the 110 respondents who has experience in working with ERP systems with use of big data technologies, 36% belong to the sector of information technology such as information systems service providers. As indicated in Table 5, the remainder is led by finance and insurance sector occupying 10% and automotive industry by 9%, while pharmaceutical sector and food and beverage sector occupy 8% each. This indicates that the integration between big data technologies and ERP systems is mainly observed in information technology, finance and insurance, automotive, pharmaceutical and food and beverage sectors.

**Table 5:** Industry sectors using the interaction of ERP systems and big data technologies

Industry Sectors	Information technology	Finance and insurance	Automotive	Food and beverage	Pharmaceuticals	Aerospace and Défense	Construction	Transportation	Home improvement and furnishing	Mining	Plastics and petrochemicals	Agriculture, forestry, fishing, and hunting	Education	Health care	Paper and packaging	Telecommunication
Count	39	11	10	9	9	8	6	4	3	3	3	1	1	1	1	1
Percentage	36%	10%	9%	8%	8%	6%	5%	4%	3%	3%	3%	1%	1%	1%	1%	1%

## Structural Equation Modelling (SEM)

The goal of SEM was to assess the plausibility of the conceptual model by measuring the relationships between two causes of effect, as a whole (Ohta et al., 2018). Plausibility is often assessed by the ability of the model to reproduce the observed key variables and sub variables (Li et al., 2019). Phase 1 of this study focused on; 1) identifying the factors influencing big data management and data contextualization, and 2) identifying the relationship between big data management, data contextualization and ERP responsiveness through a SLR. During the phase 2 of this study, causality modelling of the factors identified in phase 1 was converted to a path analysis, which hypothesized the causal relationships among variables by dividing into key variables and sub variables. The path analysis was determined by the conceptual model and the factorial analysis was conducted to test the relationships among the variables. The SPSS AMOS software was used for the data analysis. The reason for choosing SEM in empirically testing the conceptual model, was unlike other quantitative analysis methodologies such as hypothesis testing and exploratory factor analysis, SEM measures the validity of the model by going through a number of mathematical operations such as regression, path analysis and factor analysis. SEM has the unique ability of providing parameter estimates for relationships among unobserved variables (Su & Yang, 2010). SPSS AMOS is considered as a suitable software in SEM for this data analysis. The reason for using SPSS AMOS in SEM was, as the tool helps to bring out the optimum of the significant and non-significant variables, which helps in deriving the final model. In this study, we have conducted a generic SEM. SPSS AMOS software allows to easily use SEM to test hypothesis on complex variable relationships and gain new insights. Since we used SPSS AMOS software, ability to conduct a generic SEM which contains both the characteristics of CB SEM and PLS SEM was broadened. Figure 8 represents the results of the statistical analysis. The degree of freedom represents how many values involved in a calculation has the freedom to vary. Lesser the degree of freedom, proves how exceptionally connected the variables in the proposed model are (Deloitte, 2015; Gill, 2017). Therefore, the lower degree of freedom value of 153 confirms the validity of the model.

An exploratory factor analysis (EFA) was conducted to verify whether the proposed factor structures of the conceptual model are consistent with the actual data. The model was built on the data collected through the secondary research (SLR) where each dependant variable was tested against independent variables in different scenarios (e.g., different industry sectors). Since the model is not based on one set of variables tested against each other on the same scenario and none of the dependant variables are tested against the independent variables for their validity and reliability, use of confirmation factor analysis (CFA) was impossible. Therefore, an EFA was conducted to test the conceptual model using the validity and reliability of the dependant variables against the independent variables. EFA confirmed the factor structures are aligned with the conceptual model. Table 6 shows the factor structures and the findings.

The conceptual model tested using the software SPSS AMOS 7.0 estimated that the acceptance rate of the conceptual model as 1.734. Therefore, several statistical analytics such as regression (Table 7) to determine the impact of sub variables on key variables, squared multiple correlation (Table 8) to estimate the variance of the predictors of each key variable, total effect of matrices (Table 9) to determine the total direct and indirect effect of sub variables on key variables and covariances among the indices (Table 10) were used during the performance of SEM in order to further verify the variables and the conceptual model. Performance of the above-mentioned statistical analytics resulted in testing the standard error variance of the variables and probability of getting a critical ration among the key variables and sub variables.

**Table 6:** Results of exploratory factor analysis

Variables	Components							
	AN	CB	CP	V	R	RB	ES	AC
Intrinsic	-0.102	-0.003	-0.001	0.000	0.000	0.000	0.000	0.000
	-0.105	-0.004	-0.007	0.000	0.000	0.000	0.000	0.000
Usefulness	0.033	0.036	0.024	**0.373**	**0.387**	0.015	0.022	0.020
	0.034	0.035	0.022	**0.504**	**0.654**	0.020	0.025	0.021
Accessibility	**0.015**	**0.017**	**0.016**	0.020	0.015	0.383	0.495	0.096
	**0.025**	**0.024**	**0.017**	0.021	0.016	0.509	0.681	0.109
Data contextualization	0.113	0.408	0.644	0.528	0.468	**0.452**	**0.408**	**0.555**
	0.306	0.307	0.307	0.331	0.331	**0.259**	**0.243**	**0.225**
ERP responsiveness	0.014	0.023	0.059	0.031	0.024	0.062	**0.063**	0.061
	0.055	0.0**63**	0.059	0.024	0.024	0.052	0.053	0.051
Big Data management	0.078	0.085	0.063	0.3**19**	0.315	0.224	0.230	0.216
	0.065	0.075	0.064	0.304	0.3**19**	0.028	0.038	0.006

**Table 7:** Regression weights

			M.I	Estimate	Lower	Upper	Par Change
Intrinsic	< –-	Completeness	-0.179	0.002	0.357	0.365	-0.109
Intrinsic	< –-	Unambiguousness	-0.497	0.035	0.398	0.425	-0.119
Intrinsic	< –-	Meaningfulness	-0.41	0.125	0.254	0.301	-0.111
Intrinsic	< –-	Precision	-1.432	0.065	0.128	0.150	-0.125
Usefulness	< –-	Relevance	6.214	0.245	0.135	0.167	-0.135
Usefulness	< –-	Value adding	7.015	0.256	0.010	0.035	-0.178
Accessibility	< –-	ERP storage	5.426	0.096	0.009	0.029	0.180
Accessibility	< –-	Access rights	5.119	0.495	0.416	0.577	0.218
Accessibility	< –-	Representation barriers	4.333	0.383	0.297	0.457	0.223
Big data management	< –-	Intrinsic	-1.26	0.432	0.357	0.554	-0.242
Big data management	< –-	Usefulness	7.278	0.373	0.259	0.493	0.250
Big data management	< –-	Accessibility	8.258	0.350	0.257	0.465	0.232
Data contextualization	< –-	Comparison	18.742	0.062	0.291	0.086	0.390
Data contextualization	< –-	Trend evaluation	-1.742	0.002	0.091	0.080	0.090
Data contextualization	< –-	Analysis	44.020	0.116	0.138	0.368	0.929
Data contextualization	< –-	Correlation building	46.166	0.007	0.205	0.225	0.755
ERP responsiveness	< –-	Big data management	4.030	0.853	0.550	1.282	0.375
ERP responsiveness	< –-	Data contextualization	24.166	0.369	0.278	0.453	0.334

**Table 8:** Squared multiple correlation

Parameter	Standard error (SE)	Mean	Bias	SE-Bias
Usefulness	0.070	0.537	0.003	0.002
Accessibility	0.038	0.741	0.007	0.001
Big data management	0.099	0.380	0.012	0.003
Data contextualization	0.086	0.450	0.026	0.003
ERP responsiveness	0.079	0.150	0.014	0.002

**Table 9:** Total effect of matrices

	AN (Analysis)	CB (Correlation building)	CP (Comparison)	V (Value Adding)	R (Relevance)	RB (Representation Barrier)	ES (ERP storage)	AC (Access rights)	Usefulness	Accessibility	DM (Big data management)	DC (Data contextualization)
Usefulness	0.000	0.000	0.000	0.061	0.044	0.000	0.000	0.000	0.000	0.000	0.000	0.000
Accessibility	0.000	0.000	0.000	0.000	0.000	0.040	0.042	0.059	0.000	0.000	0.000	0.000
Big data management	0.000	0.000	0.000	0.083	0.078	0.035	0.044	0.012	0.182	0.090	0.000	0.000
Data contextualization	0.126	0.111	0.108	0.000	0.000	0.000	0.000	0.000	0.000	0.000	0.000	0.000
ERP responsiveness	0.066	0.054	0.107	0.041	0.039	0.006	0.007	0.002	0.106	0.015	0.127	0.158

**Table 10:** Covariances among the indices

			M.I	Par Change
Correlation building	< – >	Analysis	32.857	0.303
Relevance	< – >	Analysis	6.994	0.119
Relevance	< – >	Correlation building	10.319	0.182
Representation barrier	< – >	Correlation building	7.590	0.186
Representation barrier	< – >	Comparison	6.451	0.139
ERP storage	< – >	Correlation building	4.970	0.156
Access rights	< – >	Comparison	4.646	0.101
Access rights	< – >	Value Adding	7.503	0.128
Access rights	< – >	ERP storage	15.250	0.238
error15	< – >	ERP storage	5.426	0.066
error15	< – >	Access rights	5.119	0.053
error18	< – >	Value Adding	7.278	0.097
error18	< – >	error15	4.463	-0.038
error17	< – >	Representation Barrier	24.166	0.205
error17	< – >	error15	6.458	0.043
error19	< – >	Analysis	44.020	0.351
error19	< – >	Correlation building	46.166	0.452

Regression weights concluded the relationship between the dependant variables (i.e., key variables) with the independent variables (i.e., sub variables). According to findings, sub variables completeness, unambiguousness, meaningfulness, and precision have negative effects on the mediation variable intrinsic. Presumably, when the intrinsic factor increases by 1; the sub variables of the intrinsic factor reduce by -0.179, -0.497, -0.41 and -1.432 respectively (see Table 7). Similarly, one sub variable (i.e., trend evaluation of the key variable data contextualization) has a negative effect on it. Rest of the sub variables have positive effect on the respective key variables.

The squad multiple correlation values determine that the model is not considerably bias and the paths through the key variables and sub variables are positive and significant except for the five sub variables completeness, unambiguousness, meaningfulness, precision, and trend evaluation.

## Discussion

The main objective of this study was to investigate the factors influencing big data management and data contextualization and to identify the relationship between big data management, data contextualization and ERP responsiveness.

The conceptual model developed through SLR indicated that higher level of intrinsic values enhances big data management. As stated in Table 7, the model fit value of intrinsic factor on big data management is -1.26, which shows a negative effect on big data management. Similarly, standardized regression weight of -0.242 between the intrinsic factors and big data management concludes that if intrinsic increases by 1 standard deviation, the big data management will have a negative effect of 1.26. Through the squared multiple correlation, it shows that the variables are directly predicting intrinsic factors for an amount of 43.2% of the variations. In other words, the error factor of intrinsic variable is 36.4%. Thus, it is evident that intrinsic factors have a negative impact on the big data management. Therefore, hypotheses H1a, H1b, H1c and H1d were rejected. This indicates that the ‘completeness’, ‘unambiguousness’, ‘meaningfulness’ and ‘precision’ are not the most simplistic and essential elements in big data management. Although Gupta et al. (2018) argued that intrinsic factors such as completeness, unambiguousness, meaningfulness and precision have positive impacts on big data management, this research explained that the intrinsic factors have a negative impact on the big data management.

The model fit value of the usefulness factors on big data management is 0.250, which concludes that usefulness factors have a considerable effect on the big data management. The regression weights of the model estimate if usefulness increases by 1, the big data management will increase by a 7.278. Similarly, the model determines that the variables are directly predicting the usefulness factors for an amount of 37.3%. The model verifies that the key variable usefulness has no negative effect on any of the other variables, which ultimately concludes that usefulness is a beneficial variable for the model in determining the relationship between big data management and ERP responsiveness. Therefore, the hypotheses H2a and H2b were accepted. This indicates that the usefulness of the data is high when the collected data is relevant and value adding. Despite the fact that the relevance and value adding are appearing to be much generalized factors, the impact seems to make a considerable difference in ERP resilience. This is in line with Mukherjee and Kar (2017) and Correia and Água (2021), who have also highlighted the importance of relevance and value adding data in the context of ERP systems.

The accessibility factors which are having a model fit of 8.258 is estimated to have an amount of 38% standard error above zero along with a standardized regression weight of 0.232 in effect with big data management. Similarly, the model confirms that the key variable accessibility is having a total effect on big data management, while it does not maintain any negative effect on any of the other variables. Therefore, by considering the positive effect of accessibility factor including the sub variables, the H3a, H3b and H3c hypotheses were accepted. This indicates that accessibility mediates positive effect of access rights on big data management, accessibility mediates positive effect of data storage on big data management and accessibility mediates positive effect of representation barriers on big data management. Our findings support the discussions of Davenport (1998), Plex (2014), Shi and Wang (2018) and Zhezhnych and Tarasov (2018) by highlighting the importance of maintaining access rights, ERP storage and representation barriers.

Data contextualization is the core variable affecting the ERP responsiveness. The data contextualization variable has a direct predicting effect of 43.7% by the sub variables. The model fit confirms that data contextualization has an effect 24.16 on the ERP responsiveness. There is no standardized direct or indirect negative effect on any of the variables by the key variable data contextualization. But the sub variable trend evaluation has a negative effect on the key variable; data contextualization along with a negative model fit. Thus, it is evident that data contextualization has a positive impact on ERP responsiveness, without the sub variable trend evaluation. Therefore, the hypotheses H4a, H4c and H4d were accepted, while hypothesis H4b being rejected because of the proven negative effect on the model. This indicates that comparison, correlation building and analysis of the collected data enhance the need for data contextualization, which ultimately enhances the ERP responsiveness. Babu and Sastry (2014) and Eine et al. (2017) have also highlighted the importance of data contextualization for enhancing ERP responsiveness. The model fit value of big data management on ERP responsiveness is 0.08, which concludes that big data management have a considerable effect on the ERP responsiveness. The regression weights of the model estimate that; if big data management increase by 1, the ERP responsiveness will increase by a 24.16. Similarly, the model verifies that big data management has no negative effect on ERP responsiveness. Therefore, the hypothesis H5 was accepted. Our findings support Babu and Sastry (2014) and Eine et al. (2017), by explaining that big data management and data contextualization enhance ERP responsiveness.

**Fig. 7:**
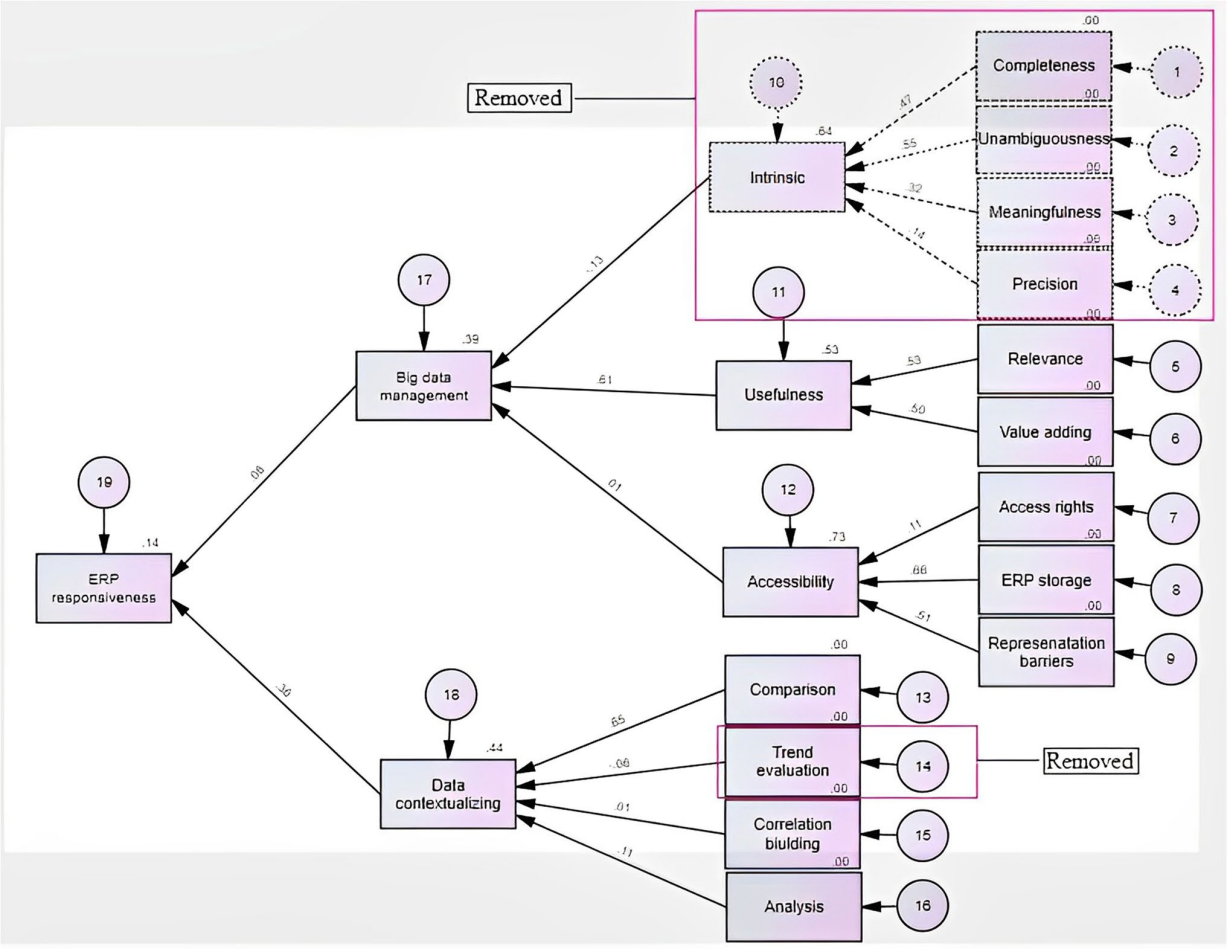
The change from conceptual model to finalized model

Figure 7 illustrates the variables that were removed from the conceptual model after conducting the empirical analysis. Based on the empirical findings as mentioned above, the variables completeness, unambiguousness, meaningfulness, and precision were removed, as a result intrinsic variable was also removed. Moreover, trend evaluation variable was also removed based on the above empirical analysis.

**Fig. 8:**
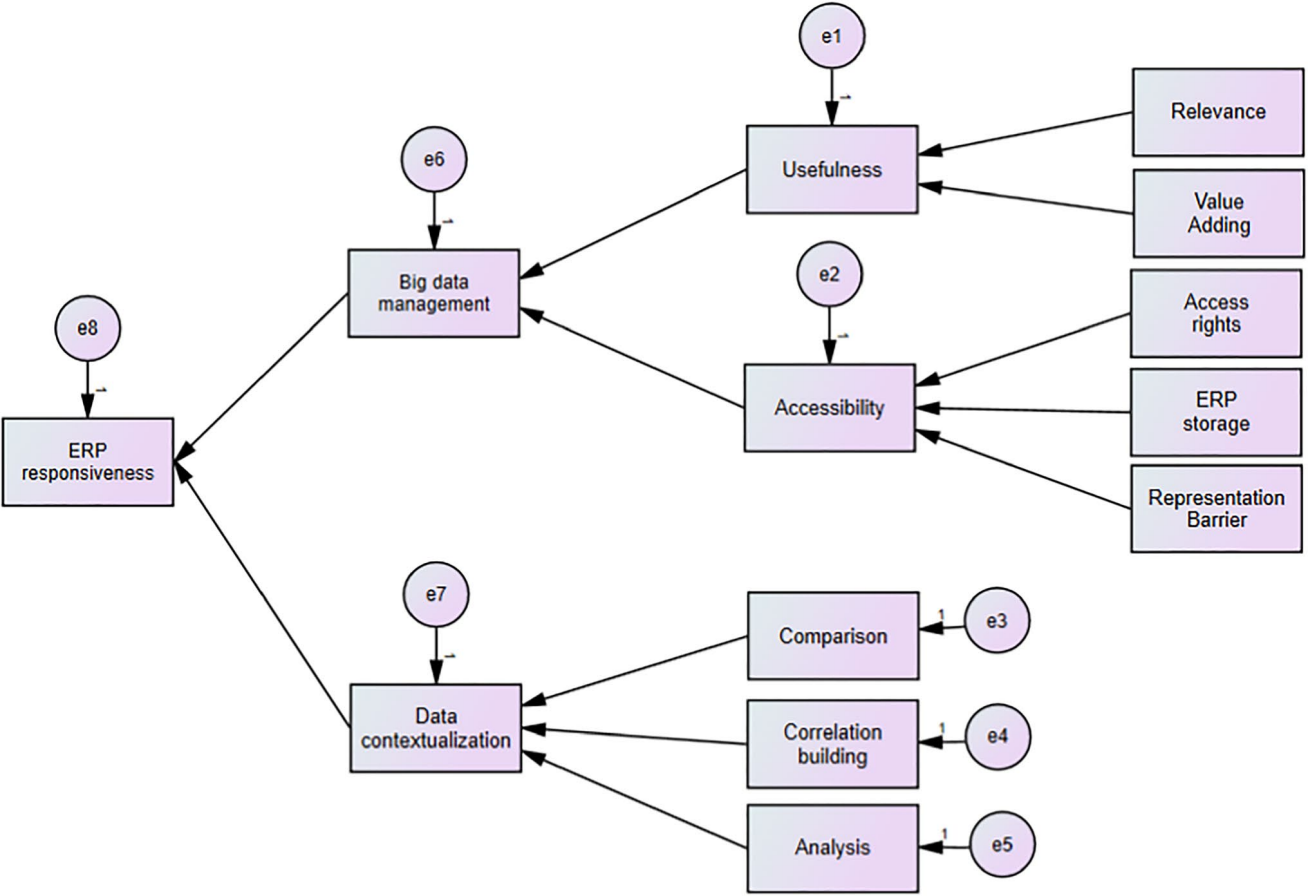
Finalized model—emerging interactions between ERP and big data

The empirical results provided a strong overall validation for the conceptual model. Reliability is about the method's consistency, and validity is about its accuracy (Schlichter & Rose, 2013). We have proved the consistency of SEM for the set of data collected in this study, as presented in Section 4.2.2. The accuracy of the findings has been demonstrated in Section 4 with the data tables from Table 6 to Table 10. The validity check of the conceptual model was conducted on construct, content, criterion, and the face, which further confirmed the reliability of the empirical results of this study. Hence, the conceptual model was modified and finalized into the model indicated in Fig. 8.

The objectives of this study were to identify the factors influencing big data management and data contextualization and to investigate the relationship between big data management, data contextualization and ERP responsiveness. A conceptual model which illustrates those factors and relationships was developed through a SLR. The conceptual model was then tested using SEM performed on the survey data collected from 110 industry experts. The results suggested factors (e.g., big data management, data contextualization, usefulness and comparison) and their relationships which impact on ERP responsiveness.

## Theoretical and Practical Implications

This paper provides empirical justification of a model which identifies; 1) the factors influencing big data management and data contextualization, and 2) the relationship between big data management, data contextualization and ERP responsiveness. It confirms that ERP responsiveness can be enhanced by big data management and data contextualization.

Gupta et al. (2018) argues that intrinsic factors including completeness, unambiguousness, meaningfulness and precision have positive impacts on big data management. However, this research revealed that the intrinsic factors have a negative impact on the big data management. Huang and Handfield (2015) and Demyanova et al. (2018) describe the factors affecting data contextualization as correlation building and analysis. This research study proposes another factor to the list as comparison, which also makes a greater impact on increasing ERP responsiveness.

This research explains usefulness variable with two sub variables as relevance and value adding, which ultimately enhances the understanding of ERP responsiveness. Previous research (for example, Mukherjee and Kar (2017), Correia and Água (2021) and Huang et al. (2008)) has also highlighted the importance of relevance and value adding data in the context of ERP systems. Granting access rights, maintaining ERP storage and implementing representation barriers improve the accessibility of the data. Similar to our findings, Shaqrah and Alzighaibi (2021) and George et al. (2014) have also explained that the organizations consider big data management due to the need to maintain relevant and value adding data. Similarly, increasing the data validity can minimize ERP and data integration failures. Previous research (Davenport, 1998; Plex, 2014; Shi & Wang, 2018) explained that maintaining relevant and value adding data can minimize the data redundancy and duplication in ERP systems.

Poor data accessibility is a common issue faced by many organizations. Schlichter and Rose (2013) presents access rights, ERP storage and representation barriers as accessibility factors. Maintaining proper access rights, keeping track on the ERP storage and maintaining quality of the representation barriers have a great impact on big data management of an ERP system. Poppe et al. (2015) and Abouelmehdi et al. (2018) have also highlighted that the necessity of securing and sharing data while maintaining access rights has led to big data management. Maintaining an adequate amount of data storage with a careful consideration of the security measures, implementing a strong set of access rights and weakening representation barriers enhance the necessity of big data management (Davenport, 1998; Plex, 2014; Shi & Wang, 2018; Zhezhnych & Tarasov, 2018).

Data contextualization is observed to have an effect on ERP responsiveness. Complexity occurs in manipulation of data in the ERP systems which influences on the responsiveness and the mobility. Thus, it is necessary for the ERP systems to have the ability to compare the data collected by the ERP systems, build a correlation among the data so as to verify if the data is needed to be collected and maintained by the ERP systems (Barth & Koch, 2019). Moreover, it is important to identify whether the collected data is having any causality among modules to reduce the data duplication and to engage the data in the analytical process to gain various outputs (e.g., predict the future of the business and analyze the profit and loss margins). Comparative nature of an ERP system is one of the core factors in minimizing data duplication and data redundancy. Developing ERP systems’ analytical abilities to build the correlation among the clusters of data, compare and analyze the data, strengthens the overall functionality of ERP systems (Li et al., 2019). Poor data management is a common issue faced by many organizations. Maintaining big data sufficiently, while keeping track of the access rights, storage, quality of the representation barriers in order to increase the usefulness, have a great impact on ERP responsiveness (Barth & Koch, 2019). Previous research (for example, Babu and Sastry (2014) and Eine et al. (2017)) has also highlighted the importance of data contextualization for enhancing ERP responsiveness.

Due to the increased amount of data collected through ERP systems, it is important that the organizations have a proper understanding on ERP responsiveness. The finalized model of this study can be used as source of guidance to be used when planning an ERP implementation. Moreover, the model indicates the factors influencing big data management and data contextualization, and 2) the relationship between big data management, data contextualization and ERP responsiveness. The model developed through this study will be helpful for managers in understanding the relationships between ERP systems and big data management. Furthermore, the model can be used as a guidance to enhance ERP responsiveness, which ultimately may minimize ERP and big data integration failures.

## Limitations and Future Research

The study developed a model which indicates the factors influencing big data management and data contextualization, and the relationship between big data management, data contextualization and ERP responsiveness. Even though this research provides rich insights to the phenomenon of study, there are a few limitations to be noted. The model can be used by the industry professional in identifying the factors impacting ERP responsiveness. Nonetheless, there can be variations based on the industry type of the organization. Future research is needed to apply the model to various industries and explain how the results varies as per the industry type. For example, as per the findings of the questionnaire, the automotive industry uses the interaction between ERP systems and big data technologies not only in business functions, but also in developing innovative car concepts. Thus, future research can explore the relationship between big data technologies and ERP responsiveness, specifically in the context of automotive industry. The study is mostly concentrated on the facts to be considered in deploying ERP systems that can deal with big data technologies during the time of planning the implementation. The participants of the questionnaire were mostly accumulated in the European continent, yet the results can be applied to other regions as majority of the participants were employed in multi-national companies. However, further collecting data from the other regions may enhance the generalizability of the model. Based on the limited number of past studies on the integration between ERP systems and big data technologies, there were no key themes on different types of analytics (such as predictive analytics) emerged through the secondary data in ‘ERP and big data’ domain. This can be an interesting phenomenon for future research.

## Appendix A The Questionnaire

### Eligibility Questions

1. I have worked and/or have a sound knowledge on Enterprise Resource Planning (ERP) systems
2. I have worked and / or have a sound knowledge on big data technologies

### General Questions

1. Select the most recent and the relevant company you have been working with use of ERP systems and Big Data technologies
  a Automotive
  b Pharmaceuticals
  c Aerospace and Défense
  d Aeronautics
  e Construction
  f Home improvement and Furnishing
  g Food and Beverage
  h Metal working
  i Paper and packaging
  j Plastics and Petrochemicals
  k Electronics and technology
  l Health care
2. What is the duration of your experience in working with ERP systems and Big Data technologies? Less than a year
  a 0—2 years
  b 2—5 years
  c 5—7 years
  d 7—10 years
  e 11 years and above
3. How many employees been working in the company you took into consideration during this questionnaire?
  a Less than 50 employees
  b Less than 250 employees
  c 250 Employee or more
4. What is the Annual turnover of the company you have been considering during this questionnaire?
  a Less than 12 million USD
  b Less than 61 million USD
  c 61 million USD or more
5. Which of the following brands of ERP systems have you been using?
  a SAP
  b SAGE
  c Oracle
  d Microsoft dynamics
  e Infor
  f Epicor
  g intuit QuickBooks
  h Other
6. Which of the following Big Data technologies have you been using?
  a Apache Hadoop
  b Apache Spark
  c R
  d NoSQL
  e Predictive Analytics
  f Blockchain
  g mongo DB
  h Cassandra
  i Other

### Questions Related to ERP Responsiveness and Big Data Management

**Table:**

		Strongly Disagree	Disagree	Neutral	Agree	Strongly agree
7	Incomplete Data makes a considerable negative impact on the ERP system's final outcome					
8	“Completeness of data” collected by an ERP system is a crucial factor in determining the success of data management by an ERP system					
9	Irrespective of the size of the company, the impact caused by incomplete data to the ERP systems are massive when it comes to decision making					
10	Mixing up of different data (unambiguous) within the ERP systems be a problem to data management of the company as an ERP system is a centralized system with a transparency					
11	In-order to increase the performance of the ERP systems, unambiguousness nature of the data collected is a must					
12	Data collected by a module can be a meaningless data to another module, creating problems to the entire company					
13	Decreasing the confusions arisen with the data is a core curriculum in maintaining big data properly					
14	Proper data mapping is a must, in-order to maintain an accurate procedure of Big Data management					
15	ERP systems only collect relevant data that is useful for all the modules					
16	Significance of the data collected and generated by the ERP systems can change with the time					
17	Increasing the validity of data collected by an ERP system is having a direct influence on the performance on data management					
18	Strictly handling access rights in an ERP system is a major factor affecting its data management					
19	Monitoring access reviews and string password hygiene are some of the measures that can be strictly maintained in order to manage Big Data in ERP systems					
20	Making use of "identity tracking software" merged to the ERP systems is an advanced measure taken to increase the interoperability with Big Data					
21	ERP systems do not act as data repositories anymore					
22	Data storage space of an ERP system is affected for its analytical performance					
23	Increasing the security of the ERP storage is a major factor affecting the ERP responsiveness					
24	Choosing of ERP systems rely mainly on the factor of "data representation"					
25	There is an influence on ERP responsiveness by Big Data Management					
26	Other than Big Data management, ability of data contextualizing is having a greater effect on ERP Responsiveness					
27	Comparison of data between modules and reacting accordingly is a crucial factor of an ERP system					
28	Trend evaluation has a bigger impact in increasing the responsiveness of ERP systems					
29	Evaluation of the current trend of the business is one of the core services performed by the ERP systems					
30	Building up of causality among the modules in an ERP system is the only way to develop the performance of an ERP system					
31	Big data technologies such as Hadoop are better to use in order to uplift the ERP responsiveness through data contextualization by increasing the analytical ability					
32	Maintaining a proper data management and increasing the ERP responsiveness are having a direct influence on improving the trust among the users					
